# Modelling Translation Initiation under the Influence of sRNA

**DOI:** 10.3390/ijms131216223

**Published:** 2012-11-30

**Authors:** Fabian Amman, Christoph Flamm, Ivo Hofacker

**Affiliations:** Institute for Theoretical Chemistry, University Vienna, Währingerstraße 17, 1090 Vienna, Austria; E-Mails: xtof@tbi.univie.ac.at (C.F.); ivo@tbi.univie.ac.at (I.H.)

**Keywords:** sRNA, sRNA target prediction, translation initiation

## Abstract

Bacterial small non-coding RNA (sRNA) plays an important role in post-transcriptional gene regulation. Although the number of annotated sRNA is steadily increasing, their functional characterization is still lagging behind. Various computational strategies for finding sRNA–mRNA interactions, and thus putative sRNA targets, were developed. Most of them suffer from a high false positive rate. Here, we present a qualitative model to simulate the effect of an sRNA on the translation initiation of a potential target. Information about the ribosome–mRNA interaction, sRNA–mRNA interaction and expression information from deep sequencing experiments is integrated to calculate the change in translation initiation complex formation, as a proxy for translational activity. This model can be used to post-evaluate predicted targets, hence condensing the list of potential targets. We show that our translation initiation model, under the influence of an sRNA, can successfully simulate thirteen out of fifteen tested sRNA–mRNA interactions in a qualitative manner. To show the gain in specificity, we applied our method to a target search for the *Escherichia coli* sRNA RyhB. Compared with simple target prediction without post-evaluation, we reduce the number of targets to less than one fourth potential targets, considerably reducing the burden of experimental validation.

## 1. Introduction

Bacteria’s competence to adapt to changing environmental conditions is one key to their ecological success. Beside the network of transcription factors, a second layer of regulation has attracted attention since 1984 when the influence of the RNA MicF on the expression of ompF was discovered [1]. Since trans-acting small non-coding RNA (sRNA) shifted into the focus of research, remarkable progress was made describing new sRNA genes in a number of bacterial species. Experimental approaches (micro-arrays, co-purification, and more recently, next generation sequencing) could successfully verify more than 80 sRNA genes in *Escherichia coli*[2]. Computational screens based on sequence conservation, structural homology or expected components, like promoters and terminators, suggest the existence of hundreds more [3]. Meanwhile the functional description of newly found sRNA genes becomes the main obstacle in broadening the existing gene regulation networks.

Functional characterization is still a challenging task. It is not clear from the outset by which mechanism an sRNA works. They bind to proteins, altering their activity [4], or they bind to target mRNA, thus influencing their stability or translation. The latter can be performed in different ways. Some sRNA block translation initiation by competing with the ribosome binding site (RBS) of the mRNA. This leads to reduced translation, which can again cause degradation of the unused mRNA molecule. A less frequent effect of a bound sRNA is to fortify the translation rate by inducing a refolding of the translation initiation region (TIR) and thus dissolving translation inhibiting structures. Additionally, some sRNA exclusively regulate only one target whereas others can interact with dozens of targets, applying a different one of the above-mentioned mechanisms each time. In contrast to miRNA in eukaryotes, where a lot of binding rules are marked out (such as a 5′ binding seed or a preference for binding sites at the ends of 3′ UTR [5]), the interactions of sRNA with their mRNA counterparts show a striking variability in bacteria [3].

All this complexity is reflected by the fact that there is no satisfying standalone technique to find new targets for an sRNA yet. Experimental approaches are very labor intensive, which means that they are not applicable to broad genomic screens (e.g., two-plasmid reporter gene assay [6]), or they are not suitable to properly distinguish between primary and secondary regulation effects (e.g., sRNA over-expression or deletion with downstream transcriptome profiling [7]).

Computational target prediction methods have shown to be helpful. The applied techniques range from mere sequence-based methods comparable with Blast [8] (e.g., TargetRNA [9]), to more sophisticated methods that calculate the hybridization energy by considering the inter-molecular base-pairing and stacking energies (implemented in, e.g., RNAduplex, part of the ViennaRNA Package [10]). The latest generation also includes intra-molecular structure, thus taking the accessibility of the putative binding site into account. This approach was implemented in RNAup [11], IntaRNA [12] and most recently RNAplex [13] in combination with RNAplfold [14,15]. The structure based tactics are similar in their attempt to find the best possible interaction or interactions between two given RNA sequences. Since any two sufficiently long sequences will show some stable interaction, the decision of which sequences to search and how the results are interpreted is up to the user. A common strategy is to concentrate on a sequence stretch of −30 nt to +20 nt around the translation start site [9], which, by reducing the search space, reduces the number of predicted nonfunctional binding sites. This strategy has proven to be quite successful since many observed interactions are indeed taking place in this region. However, some interactions are known to be further upstream. In *E. coli*, DsrA and RprA bind their target rpoS at position −94 nt and −93 nt, respectively, upstream of the translation start site where they induce an activation of translation [16]. OmrB represses csgD by binding from position −79 nt to −61 nt in front of the gene’s start site [17]. Even in the reduced search space around the start codon, it seems that the thermodynamically best binding sites are not always the biologically functional ones. Some experimentally observed binding sites show an unfavorable calculated binding energy and thus are easily overseen in genome wide screens. This might be explained by the activity of chaperons such as Hfq, which stabilize the sRNA–mRNA interaction [18].

This is why we developed a new approach to extend the common binding site prediction with an automated evaluation of the functional consequences of a bound sRNA on translation initiation. This is achieved by introducing a model that simulates the initiation of translation in the system mRNA, sRNA and 16S ribosome. With this approach, it is possible to examine which of the putative interactions have the potential to interfere with translation initiation. In the following article, we will lay out how our model can simulate this influence and show that this can be helpful to evaluate predicted target sites for their biological significance.

## 2. Model Description

Translation initiation is the process by which components of the ribosome detect an mRNA, which leads to the assembly of the ribosomal machinery. It was demonstrated that this is the rate limiting step for translation [19]. It is triggered by the binding of the 30S ribosome unit, via the 3′ end of the 16S ribosomal RNA, to the Shine–Dalgarno sequence (SD) and the positioning of the fMet-tRNA*^fMet^* anti-codon to the correct start-codon on the mRNA. A mathematical model of this process was developed by Na and Lee [20], whose concept and nomenclature are adopted here. The model was slightly adapted and substantially expanded to include the influence of sRNA binding on translation initiation.

Kinetically, the initiation of the ribosome–mRNA interaction is driven by the energy gained from the hybridization of the 16S rRNA to the ribosome recognition site (RRS, *i.e*., a generalization of the Shine–Dalgarno sequence) and the anti-start-codon–start-codon interaction. Further on, the accessibility of the complete ribosome docking site (RDS, *i.e*., the stretch of the mRNA that is occupied by the translation initiation complex) is essential because during initiation the ribosome has no capability to dissolve inhibiting structures on the mRNA [21]. At this point, the sRNA can interfere with ribosome binding: Either it competes with the ribosome for binding within the RDS or it alters the accessibility of the RDS by binding close-by and inducing a refold, hence changing the mRNA accessibility for the ribosome.

We define the RRS as the energetically most favorable binding site of the anti-RRS (the 3′ end of the 16S rRNA, in the case of *E. coli* this would be “UCACCUCCUU”) upstream of the translation start site. Calculating all possible interactions and choosing the energetically most favorable one, provides the position of the RRS and the ribosome–mRNA hybridization energy Δ*G_R_*. To account for the stabilizing effect of anti-start-codon–start-codon interaction, −1.19 *kcal*/*mol* for *AUG*, −0.075 *kcal*/*mol* for *GUG* and 0 *kcal*/*mol* for all other are added to Δ*G_R_*[22].

The RDS was shown to be about 30 nt long [19], starting from the predicted RRS start. The RDS exposing probability of the free mRNA *P_EF_* (*i.e*., the probability that this 30 nt long sequence is accessible for the ribosome), or equivalently the free energy Δ*E_F_* = −*RT* ln *P_EF_* needed to make the RDS accessible, is the main thermodynamic barrier in translation initiation.

Regarding the system consisting of mRNA, sRNA and ribosome, the following reactions (Equations 1–4) lead from the free unbound mRNA *M_F_* to the ribosome bound mRNA *M_R_* or can compete with this reactions. For simplicity, Equation 4 itself is not included in the model.

(1) $$M_{F}+S_{F}\overset{K_{S}}{⇌}M_{S}$$

(2a) $$M_{F}^{*}+R_{F}\overset{K_{R}}{⇌}M_{R}$$

(2b) $$M_{F}\overset{K_{EF}}{⇌}M_{F}^{*}$$

(3a) $$M_{S}^{*}+R_{F}\overset{K_{R}}{⇌}M_{SR}$$

(3b) $$M_{S}\overset{K_{ES}}{⇌}M_{S}^{*}$$

(4) $$M_{R}+S_{F}\overset{K_{SR}}{⇌}M_{SR}$$

Thereby, *M_F_* is the free unbound mRNA, *R_F_* the free ribosome, *S_F_* the free sRNA, *M_S_* and *M_R_* the sRNA and the ribosome bound mRNA, respectively. *M_SR_* represents the mRNA species with sRNA and ribosome bound at the same time. The superscript asterisk “*” marks the RDS exposing fraction of its kind. In the following, we will use the convention to address reaction species with uppercase letter, whereas lowercase letters are used when we refer to the concentration of the particular reaction species.

The equilibrium constants of the ribosome binding and sRNA binding reaction, $K_{R}=\text{exp}\left(-\frac{\Delta G_{R}}{RT}\right)$ and $K_{S}=\text{exp}\left(-\frac{\Delta G_{S}}{RT}\right)$, respectively, can be calculated from the free energy difference of the reaction Δ*G_R_* and Δ*G_S_*, where *T* is the temperature and *R* the gas constant. Please note that the reaction constant for the ribosome binding to the mRNA *K_R_* is independent of mRNA structure, thus the same in Equation 2a,3a. The mRNA structure is already considered through the formation of M* (Equation 2b,3b).

*K_EF_* and *K_ES_* denote the equilibrium constants of the unfolding reaction of the complete RDS, without and with the influence of a bound sRNA, respectively. The reaction constants are connected to the probabilities *P* to expose the RDS by $P=\frac{K}{1+K}$. In the following we will only work with the corresponding probabilities *P_EF_* and *P_ES_*.

To calculate the amount of ribosome bound mRNA, the relative positions of the sRNA binding site and the RDS have to be considered. In the case where the RDS overlap with the sRNA binding site, reaction 3a is not possible since a simultaneous binding of the ribosome and the sRNA is sterically not possible, thus species *M_SR_* does not occur. If RDS and sRNA binding site are spatially separated, sRNA and ribosome can bind to the same mRNA molecule, hence two translational active mRNA species, *M_R_* and *M_SR_*, have to be considered.

**Figure 1:**
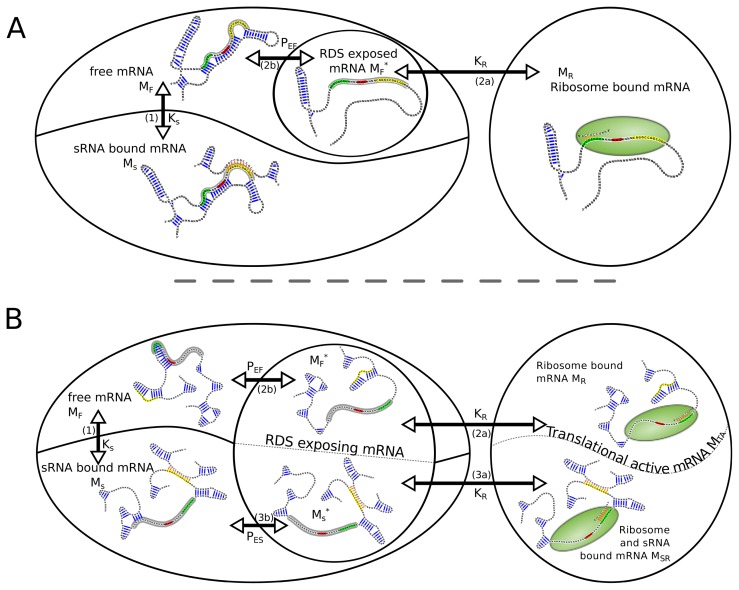
Graphical illustration of all reactions and species considered in the reaction network. The RNA species are depicted with black backbones, blue intra-molecular and orange inter-molecular base-pairs. The ribosome with its anti-RRS sequence is shown as a green sphere. The RDS is highlighted in gray. The RRS, the start codon and the RNA binding site are marked with green, red and yellow, respectively. Reactions are symbolized with *↔* arrows, their corresponding equilibrium constants and a reference to the reaction equation in the main text. (**A**) In the case where the RDS and the RNA binding site overlap, two reaction branches from *M_F_* compete with each other. One leads to sRNA bound mRNA *M_S_*, the other leads via M_F_^*^ to ribosome bound mRNA *M_R_*; (**B**) In the case where the RNA binding-site and RDS are spatially separated, there are two routes from free mRNA to translationally active *M_TA_*. One leads as before via M_F_^*^ to *M_R_*. The other route first leads to an sRNA·mRNA complex, which can further expose its RDS M_S_^*^, and eventually ends in the active ribosome·mRNA·sRNA complex *M_SR_*.

The chemical reaction network above can be readily translated into a system of equations describing the equilibrium concentrations of all chemical species. In the following, we use this to calculate the amount of ribosome bound mRNA and its dependence on sRNA presence. Figure 1 depicts the different routes and reactions that lead from the unbound mRNA to translational active, namely ribosome bound mRNA.

### 2.1. Overlap of sRNA-BS and RDS

Since sRNA and ribosome cannot bind the same mRNA, the only translational active mRNA is the *M_R_* species. The ribosome binds the free RDS exposing mRNA in thermodynamic equilibrium with

(5) $$K_{R}\;m_{F}^{*}\;r_{F}=m_{R}$$

At the same time the sRNA binding competes with this reaction. sRNA binding onto free mRNA can be described with

(6) $$K_{S}\;m_{F}\;s_{F}=m_{S}$$

Furthermore, the following relationships can be formulated, thereby *s_F_*, *s_T_*, *m_F_*, *m_S_*, *m_R_*, *m_T_*, *r_F_* and *r_T_* describe the concentrations of free sRNA, total sRNA, free mRNA, sRNA bound mRNA, ribosome bound mRNA, total mRNA, free ribosome and total ribosome, respectively.

(7) $$s_{F}+m_{S}=s_{T}$$

(8) $$m_{F}+m_{S}+m_{R}=m_{T}$$

(9) $$r_{F}+n\;m_{R}=r_{T}$$

The pool of free ribosomes is depleted not only by ribosomes bound at the TIR but also by actively translating ribosomes. To account for this, we follow Na and Lee [20] and introduce the ribosome occupancy *n* in Equation 9. The value *n* is estimated from experiments on the *E. coli* lac operon that show on average 20 ribosomes bound to the mRNA [23]. Thus, each initiation event (as modelled by Equation 5) ultimately reduces the number of free ribosomes by approximately *n* = 20.

Taking this system of five equations (Equations 5–9) together with m_F_^*^ = *P_EF_m_F_* allows to compute the amount of translation initiation complex *m_R_* as function of *K_R_*, *K_S_*, *P_EF_*, *s_T_*, *r_T_*, *m_T_* and *n*. In principle the variables *s_F_*, *m_F_*, m_F_^*^, *r_F_* and *m_S_* can be eliminated resulting in a cubic polynomial that is analytically and numerically solvable. Details can be found in the supplementary material.

### 2.2. No Overlap of sRNA-BS and RDS

When RDS and sRNA binding site are spatially separated, both binding sites can be occupied at the same time. As a consequence, two species in the described reaction network represent active translation initiation complexes. To contribute for this we introduce a new variable for the translational active mRNA *m_TA_*.

(10) $$m_{TA}=m_{R}+m_{SR}$$

Furthermore, we have to consider reaction 3a, describing the binding of a ribosome to an sRNA·mRNA complex

(11) $$K_{R}\;m_{S}^{*}\;r_{F}=m_{SR}$$

In contrast to the first case with overlapping RDS and sRNA-BS, Equations 7–9 have to be adapted in the following way to include the new species of *m_SR_*

(12) $$s_{F}+m_{S}+m_{SR}=s_{T}$$

(13) $$m_{F}+m_{S}+m_{R}+m_{SR}=m_{T}$$

(14) $$r_{F}+n\;(m_{R}+m_{SR})=r_{T}$$

As before it is possible to eliminate from the seven Equations 5, 6, 10–14 additional with m_S_^*^ = *P_ES_m_S_* the variables *m_F_*, m_F_^*^, *s_F_*, *r_F_*, *m_R_*, *m_S_*, m_S_^*^ and *m_SR_*. The result is a quintic polynomial equation describing the translational active mRNA *m_TA_* as a function of *K_S_*, *K_R_*, *n*, *P_EF_*, *P_ES_*, *m_T_*, *r_T_* and *s_T_*, which can be numerically solved.

### 2.3. Model Implementation

**Figure 2:**
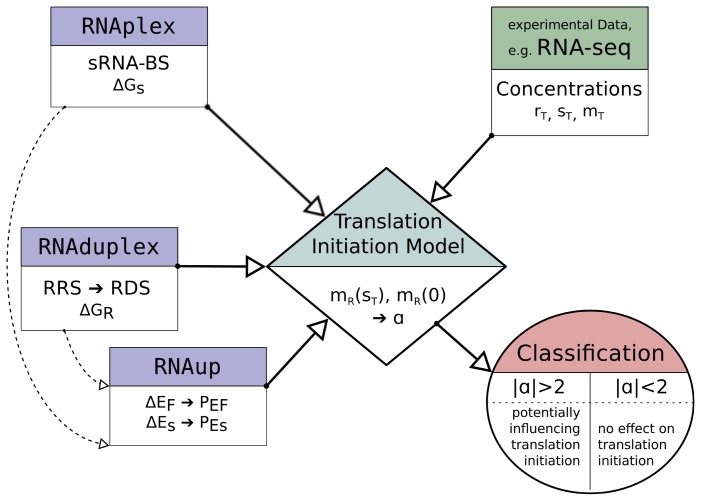
Illustration of the work-flow for the classification of whether sRNA binding can influence the mRNA’s translation initiation. RNAplex is used to calculate possible sRNA– mRNA interaction sites. RNAduplex calculates the ribosome–mRNA interaction, hence determining the position of the RRS and RDS, and the hybridization energy Δ*G_R_*. The position of the RDS and the sRNA binding site (sRNA-BS) is used with RNAup to determine the exposing probabilities *P_EF_* and *P_ES_*. The concentrations of all reactants are deduced from RNA-seq data. All this information is integrated in the *Translation Initiation Model* to calculate the amount of mRNA that is bound by the initiation complex assuming the presence (*m_R_*(*s_T_* )) and the absence (*m_R_*(0)) of sRNA. The ratio *α* of these serves as a descriptor to classify the potential of the sRNA to influence translation initiation.

The described model equations contain concentration data, *s_T_*, *m_T_*, *r_T_* and *n*, which can be deduced from experiments (e.g., RNA-seq or tiling arrays) and equilibrium constants, *K_S_*, *K_R_*, *P_ES_* and *P_EF_*, which all can be calculated. To perform this calculations and solve the equations, we developed a software-wrapper that makes extensive use of programs included in the ViennaRNA Package [10]. A more detailed description of the programs used can be found in Table 1. Figure 2 illustrates the main work-flow of the model implementation.

The potential sRNA-BS are determined with RNAplex, considering the accessibility of potential binding sites on the sRNA and mRNA. The accessibility is calculated with RNAplfold (the -W and -L parameter are set to 200 and 150, respectively [24]). All sub-optimal binding sites up to a binding energy Δ*G_S_* of −7 *kcal/mol*, which are at most 150 nt upstream to 20 nt downstream of the translation start site and at least 10 nt long (including inter-molecular bulges), are considered for follow-up evaluation of their potential to influence translation initiation. RNAplex-based target prediction results in sRNA-BS coordinates and the binding energy Δ*G_S_*, which includes (in contrast to Δ*G_R_*) the energy needed to make the binding sites accessible.

The search for the RRS is performed by RNAduplex, which calculates the energy and position of the optimal binding site between two given RNA molecules. RNAduplex only considers inter-molecular interactions. Intra-molecular base-pairs are ignored but inter-molecular bulges and internal loops are permitted [25]. The search space was set to −30 nt upstream to +3 nt downstream of the translation start site against the 10 nucleotides at the 3′ end of the 16S rRNA. This provides the position of the RRS and the corresponding hybridization energy Δ*G_R_* of the ribosome to the mRNA. From the RRS position the RDS position can be directly deduced to be *RRS_start_* to *RRS_start_* + 30 *nt*. The opening energy Δ*E_F_* of the RDS was calculated with RNAup [11], using a sequence stretch of *±* 250 nucleotides around the RDS. From the opening energy Δ*E_F_* the probability of being fully unfolded can be deduced from $P_{EF}=\text{exp}\left(-\frac{\Delta E_{F}}{R\cdot T}\right)$.

To calculate the sRNA influenced opening energy Δ*E_S_*, RNAup is used again. However, this time a constraint folding approach is applied, which prevents bases interacting with the sRNA to participate in intra-molecular folding of the mRNA. Once again, the probability *P_ES_*, that the complete RDS is unstructured, is given by $P_{ES}=\text{exp}\left(-\frac{\Delta E_{S}}{R\cdot T}\right)$.

The software wrapper is provided with the anti-RRS sequence, an sRNA sequence, an mRNA sequence with annotated translation start site and information about the concentrations of the reaction members. The reaction constants are determined as described above and fed into the corresponding equation system to solve the number of ribosome bound mRNA, hence translational active mRNA, in the presence of sRNA, *m_TA_*(*s_T_* ), and without sRNA, *m_TA_*(0). The corresponding equation is solved numerically applying Newton’s method. In the case where RDS and sRNA-BS overlap, we can set *m_TA_* = *m_R_*. For each analyzed putative sRNA binding site, the signed ratio $\alpha =\frac{m_{TA}(s_{T})}{m_{TA}(0)}$ (or $\alpha =-\frac{m_{TA}(0)}{m_{TA}(s_{T})}$ if *m_TA_*(0) *> m_TA_*(*s_T_* )) is returned as a measure of the sRNA induced change of translation initiation efficiency. We consider all mRNA whose translation initiation rate changes more than 2-fold (*|α| >* 2) to be putatively regulated by the corresponding sRNA.

## 3. Simulation of Known sRNA–mRNA Interactions

To test our model, we simulated the effect of sRNA binding onto translation initiation for several well-described sRNA and their targets. Since the distinguishing characteristic of the presented approach is the possibility to qualify the regulatory effect of a proposed sRNA–mRNA interaction, the focus was set on all sRNA in *E. coli* for which experimentally validated cases of positive regulation are known, *i.e*., DsrA, RprA, ArcZ, GlmZ and RyhB [26]. Thereby all confirmed interactions (positive as well as negative) of those sRNA were simulated (see Table 2).

The mRNA expression levels were estimated from publicly available deep sequencing data obtainable at the *Sequence Read Archive* (submission ID: SRA050648). Briefly, *E. coli MG1655* was grown in rich media, no rRNA depletion was performed prior to RNA-seq, 49,979,354 reads were produced [27]. The obtained reads were mapped onto *E. coli* genome (NC_000913), using segemehl [28] with default settings. The mapped reads were assigned to the corresponding protein coding and ncRNA genes, annotated in the Refseq database. If a read mapped *n* times equally well to the genome, we counted 1*/n* for each position. The counts for each gene were normalized for gene length and total read count (RPKM, Reads Per Kilobase of gene per Million mapped reads). The total number of 16S rRNA molecules within the cell is assumed to be 57,000 [20,23]. Thus, the RPKM values for each gene were further normalized by dividing by the sum of all seven 16S rRNA RPKM values and multiplied by 57,000. The resulting values are supposed to reflect the concentration ratios between the 16S rRNA and the mRNA molecules. 3833 genes were shown to be transcribed, 489 genes showed no transcription at all.

At any given moment, about 80% of the ribosomes are actively engaged in translation [29], thus reducing the number of total ribosome *r_T_* that are available for translation initiation to 11,400 per cell. Unfortunately, many of the sRNA are not expressed under the conditions of the RNA-seq experiment. We therefore used an ad-hoc estimate of sRNA concentrations (under conditions where the sRNA is active) and we set the ratio of sRNA and mRNA molecules to be 2*/*3. This is motivated by the idea that, presuming a similar state of the transcriptome, inducing sRNA gene expression to a level of 2*/*3 of the target gene should already yield a visible effect on the translation initiation rate of the target gene. To get a rough estimate of the scale of this ratio in bacteria, we examined all *E. coli* trans-acting ncRNA from the EcoCyc database [30] whether they were shown to be expressed in rich growth medium. Seven ncRNA genes fulfill this criterion, of which five are also described in terms of their targets (e.g., micM, mcaS, glmY, omrA and mgrR with a total of 12 targets). We calculated the $\frac{\left[sRNA\right]}{\left[mRNA\right]}$ ratios for all sRNA-target pairs from the normalized RPKM values and deduced the geometric mean of 0.84, close to our value 0.67 estimated from theoretical considerations.

To use the most realistic model of the mRNA possible, we reconstructed primary transcripts from a detailed analysis of the *E. coli* transcriptome [31], considering the experimentally validated operonic architecture and transcription start sites.

Based on this, thirteen out of fifteen experimental interactions could be modeled qualitatively correctly (Table 2). For tpx RNAplex does not find any potential ArcZ binding site *±*200 nt around the ribosome docking site that has less than or equal to −7 *kcal/mol* free energy. The calculated interaction between RyhB and shiA takes place from −59 nt to −48 nt. This is in contradiction to the binding site found experimentally, which is between position −76 nt and −27 nt upstream of the translation start site [32]. Applying this elongated binding site leads to a RDS accessibility change from *P_EF_* = 1.2*×*10^−5^ to *P_ES_* = 7 *×* 10^−4^.

## 4. Usage as Target Prediction Tool

The presented translation initiation model under the influence of sRNA binding has two new features. First, it integrates information about the transcript concentrations and the thermodynamic properties of the sRNA–mRNA and the ribosome–mRNA system. Second, it is possible to evaluate all putative binding sites for their capability to influence translation initiation. The first should be helpful in increasing the specificity, the latter should increase sensitivity, compared with existing target prediction methods.

To test the predictive power of our model, it was applied to predict all sRNA that can regulate RpoS translation, as well as all mRNA that are putatively regulated by RyhB.

### 4.1. Searching sRNA Controlling RpoS Translation

RpoS is an especially interesting gene because it was shown that it is activated by three different sRNA. RpoS is an alternative *σ* -factor that helps the RNA polymerase to recognize promoters of genes involved in stress response and secondary metabolism [33], thus making RpoS a central node in integrating information about the status of the cell. This is achieved by a variety of regulatory mechanisms on all levels. Beside, the known sRNA regulators of rpoS, it was suggested that other so far unknown sRNA may regulate rpoS translation [34,35].

All ncRNA from Refseq that are annotated neither as ribosomal nor tRNA (65 genes in total) were used to evaluate their potential effect on RpoS translation. An interaction is considered potentially functional if it causes more than *±*2 fold change *α*, takes place at −150 nt to +20 nt from the translation start, and has an interaction length of at least 10 nt and a binding energy Δ*G_S_* of at most −7 *kcal/mol*.

Six ncRNA fulfilled these criteria (Table 3). Beside the three above-mentioned known interactions, taken from [26], there are three additional sRNA genes with the potential to repress RpoS translation. All three of them have a higher, thus less favorable, hybridization energy, compared with the validated interactions. For OxyS it was already reported that oxyS over-expression decreases RpoS expression [36].

This analysis was also used to test how sensitive the results are to the chosen parameters. The same analysis was performed with different values for the ribosome occupancy (*n* = 1 to 100) and for the concentration ratios $\frac{\left[sRNA\right]}{\left[mRNA\right]}=1/2,2/3,1/1,3/2,2/1,3/1,4/1$. For all of them the same potential regulators were predicted, except for $\frac{\left[sRNA\right]}{\left[mRNA\right]}=1/2$ where only dsrA and arcZ showed the potential to influence rpoS translation.

### 4.2. Searching mRNA Controlled by RyhB

RyhB is a 90 nt long sRNA that plays an important role in cell homeostasis. Under conditions of iron starvation, RyhB is expressed and reduces the translation of non-essential iron-using proteins [37].

Potential binding sites of RyhB on all 4146 protein coding genes annotated in NCBI Refseq were calculated with RNAplex. 1921 genes, including all eight known targets, have an RyhB binding site with a binding energy Δ*G_S_≤* −7 kcal/mol in the vicinity of their translation start (−150 nt to +20 nt).

**Figure 3:**
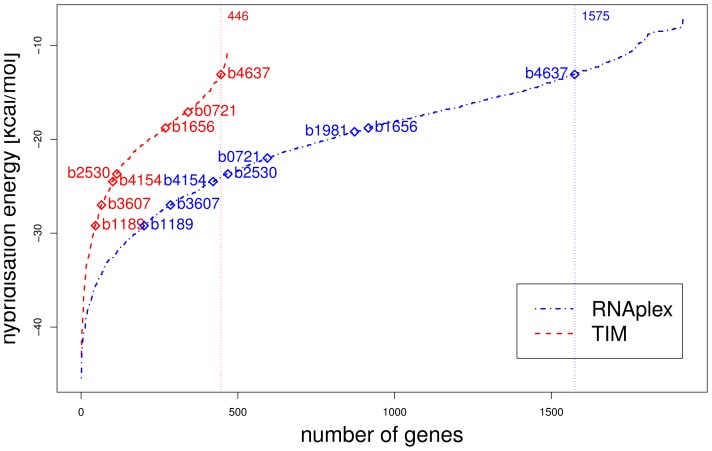
The distribution of hybridization energy. The blue curve shows the minimal hybridization energy for each gene with a calculated binding site from −150 nt upstream to +20 nt downstream of the translation start site and Δ*G ≤* −7 kcal/mol. The experimental validated genes are marked with *⋄*. In contrast, the red curve shows the hybridization energy for all genes that are potentially altered in their expression by RyhB, according to our Translation Initiation Model (TIM).

Sorting the most favorable binding sites in this neighborhood according to their hybridization energy, without post evaluation with our translation initiation model, results in the eight interactions described in literature among the 1575 most stable interactions (Figure 3).

After applying our translation initiation model and removing all interactions that seem to lack the potential to change the translation initiation rate more than *±*2 fold, only 446 binding sites had a more stable hybridization energy than the least stable known interaction (b4637 with −13.1 *kcal/mol*). In total 467 genes seemed to be potentially targeted by RyhB (Figure 3). Here shiA (b1981), a well documented activation target of RyhB, is no longer detected (see Section 3). A more detailed inspection of one particular putative RyhB target is given in the supplementary material (Section 1).

We compared the found 467 genes with experimental results from micro-array analysis with an inducible ryhB gene [38]. A general drawback of this kind of experiment is the difficulty to distinguish between directly and indirectly regulated genes. The authors tried to circumvent this by reducing the time span between RyhB induction and the assay to 15 min. This time could be still too long considering their own results for the gene exbBD, which, although most probably an indirect regulated gene, showed already after 7.5 min a significant drop in mRNA abundance. To identify genes regulated by fur, which itself is regulated by RyhB, the assay was compared with a fur^−^ mutant. In spite of this precautionary measure, the possibility that another transcription factor is RyhB controlled cannot be ruled out, hence the targets found can still be indirectly regulated by RyhB. In [38], 56 gene targets from 18 different operons could be identified as being regulated by RyhB, whereas in our analysis, in 12 out of 18 operons (~67%) we find at least one gene that is regulated in the same sense than observed in the micro-array experiment.

It is worth noting that it is not always the energetically most favorable binding site within our search region of −150 nt to +20 nt around the translation start site, which has the strongest effect on translation initiation in our model. For example, RyhB can bind sdhC (b0721) −149 nt in front of the translation start with an hybridization energy of −22.0 *kcal/mol*. According to our model, this has a negligible effect on translation initiation of *α* = +1.0007. The energetically less favorable binding site with −17.1 *kcal/mol*, which overlaps the ribosome docking site, has a significant effect of *α* = −2.87.

Although the pool of putative targets could be decreased by our binding site evaluation, 467 targets (*~*10 % of all genes) still seem implausible. At the moment, a comprehensive set of confirmed direct RyhB targets is still lacking, which would enable a detailed analysis of the specificity and sensitivity of our modeling approach. To get at least an idea of the significance of our results, we tested those 467 genes for the enrichment of certain functions described with Gene Ontology terms [39] using a web-based tool (Database for Annotation, Visualization and Integrated Discovery (DAVID) [40]). This revealed that *~*5 % of the putative targets are associated with the GO term *anaerobic respiration* and *~*10 % with the term *iron ion binding* (see Table 4). The *p*-values of this enrichment are 1.0 *×* 10^−12^ and 7.3 *×* 10^−10^, respectively. This is in perfect agreement with the role of RyhB in the cell, indicating that the regulon of RyhB is indeed much larger than the experimentally validated eight targets.

## 5. Discussion

We presented a method to evaluate the capability of predicted sRNA–mRNA interactions in interfering translation initiation. We successfully simulated the effect of five *Escherichia coli* sRNA onto their experimentally validated targets. Furthermore, we used our method to predict potential regulators of RpoS and potential targets of RyhB. The latter was compared with target prediction without post-processing. Applying our translation initiation model reduces the list of successfully predicted known targets from eight to seven. At the same time, the number of potential targets is reduced from 1921 genes to 467 genes.

A further novelty of our approach is the possibility to distinguish between translation activation and repression for the predicted sRNA–mRNA interaction. While we show the usefulness of calculated fold changes in the formation of initiation complexes (*α* values), there remain reasons to be cautious with a quantitative interpretation of *α* values. For example, our model considers only one binding site at a time, and therefore does not model the competition of several mRNA for an sRNA. Moreover, the actual kinetics might be more important than the equilibrium state, especially because of the fact that bacterial translation initiation already occurs co-transcriptional, changing the chronology of binding sites becoming available, which can drastically change the kinetic behavior of the system from the equilibrium state. Finally, translation initiation is a highly stochastic process occurring in bursts [23], which is not considered in the presented model. Considering this, we do not think that the *α* values can serve as suitable classifier to rank the reliability of predicted targets. Nevertheless, we could show that a mere binding energy based ranking leads to a significant enrichment of known targets within the top ranked genes, after evaluation of the binding sites. For the application of our model to the sRNA RyhB, there are no known targets within the top 125 ranked genes, according to a mere interaction based prediction. In contrast, after evaluating the putative interactions with our translation initiation model, we find 4 known targets within the top ranked 125 genes.

Our target prediction approach is the first to explicitly model the concentration dependence of sRNA–mRNA binding. With the advances in high throughput transcriptome quantification, such as RNA-seq or genomic tiling arrays [41], more data on mRNA expression levels are becoming available. Unfortunately, these data often do not include sRNA or are not measured under conditions relevant for sRNA regulation. We tried to find a compromise for this by deducing the concentration ratios between mRNA and ribosome from biological experiments, but assumed the ratio $\frac{\left[sRNA\right]}{\left[mRNA\right]}$ to be 2*/*3. In the near future, when more expression data for different species and different conditions will be publicly available or cheaper to produce, this problem might be overcome.

The role of the RNA chaperon Hfq is not considered in our model. Hfq is thought to enable sRNA-based translation regulation either by (1) protecting the sRNA from RNase E degradation, (2) recruiting RNase E to degrade the Hfq·mRNA·sRNA complex, or (3) facilitating the interaction between sRNA and mRNA [42]. The first would change sRNA abundance, which we avoid by assuming an effective sRNA concentration in the first place. The second mechanism, where Hfq mediates mRNA degradation, is ignored in our model which exclusively describes the sRNA effect on translation initiation. For the last mentioned mechanism, Hfq works as a chaperon, changing the kinetics of sRNA–mRNA interaction. It was shown that sRNA·mRNA complexes established this way remain stable after Hfq removal [43]. This implies that regarding the thermodynamic equilibrium state may be sufficient to detect Hfq dependent targets. An extension of our model, including effects of Hfq, is possible, but would require more knowledge about the strength and specificity of RNA–Hfq interactions.

The discrepancy in the number of confirmed interactions from biological experiments and from computational screens is puzzling. To our knowledge, the most comprehensive investigation of an sRNA regulon was published by Sharma *et al*. [44]. There, a genome-wide experimental approach and bioinformatic target prediction was combined. The regulon of GcvB in *Salmonella thyphimurium* could be enlarged to 54 genes, which corresponds to 45 different cistrons, of which 21 could be individually confirmed. We agree with the authors that this is most likely not the end of the line. Due to the fact that so far most genomic screens are solely based on changes in mRNA concentrations, which do not have to go along with translational regulation, some targets could be still missed. Furthermore, technical difficulties (e.g., read out methods) can increase the false negative rate.

Conferring this analysis to the situation of RyhB in *Escherichia coli*, together with the fact that our prediction method found 45 new targets associated with the molecular function “iron ion binding”, suggests that the regulon of RyhB is indeed much larger. Besides, it shows that our bioinformatic approach of blending RNA interaction with translation initiation is a promising tool for sRNA target prediction.

We plan to provide the described approach to the scientific community as a web-based service incorporated into the RNApredator [45] target prediction web-server (http://rna.tbi.univie.ac.at/RNApredator/) as a post-processing analysis.

## 6. Conclusions

From our point of view, computational and experimental techniques each have their advantages and disadvantages. For a complete understanding of the role of sRNA in the bacterial cell, computational and experimental biologists should rethink and enlarge their repertoire of techniques. We hope that the presented approach serves to this end.

## Supplementary File



## Figures and Tables

**Table 1:** Overview of the modules from the ViennaRNA Package used in the implementation of our translation initiation model. Manuals with more detailed descriptions can be found at www.tbi.univie.ac.at/~ronny/programs/<program_name>.html.

Program Name	Program Description	Reference
RNAduplex	Computes optimal structures upon hybridization of two R- NA strands and the free energy of the resulting duplex. The calculation is simplified by allowing only inter-molecular base pairs.	[10]
RNAplex	Finds optimal sub-optimal target sites of a query RNA on an mRNA by computing secondary structures for their hybridization. Accessibility effects are included in an approximate manner, based on accessibility profiles computed by RNAplfold.	[13]
RNAplfold	Performs local folding of very long sequences, allowing only base pairs with a maximal span of *L*. It computes mean pair probabilities as well as accessibilities for every position *i*, averaging over all sequence windows of length *W* that contain *i*. The resulting accessibility profiles can be used, e.g., in RNAplex.	[14,15]
RNAup	Computes accessibilities, *i.e*., the probability *P_u_*[*i; j*] that a sequence interval [*i; j*] is unpaired, with an extension of the standard partition function approach for RNA secondary structure. This computation can also be conducted with constraints to force specified bases to remain unpaired, which allows us to compute accessibilities with- and without bound sRNA.	[11]

**Table 2:** The modeled changes in translation initiation rate for five sRNA. *Regulation Type* gives the experimentally shown behavior of the system. *Position (mRNA)* gives the calculated site of sRNA binding onto the mRNA relative to the start codon. *Hybridization Energy* gives the energy gained by the hybridization of the mRNA and the sRNA in *kcal/mol. Fold Change α* is the resulting value, according to the simulation, how much the initiation rate changes with and without sRNA.

sRNA	mRNA	Regulation Type	Position (mRNA)	Hybridization Energy	Fold Change *α*
dsrA	hns	repression	(−12)..+18	−22.9	−2.94
	rpoS	activation	(−126)..(−97)	−33	+2.99

rprA	rpoS	activation	(−133)..(−94)	−30.7	+2.11

arcZ	rpoS	activation	(−105)..(−81)	−23.3	+13.50
	sdaC	repression	(−13)..(−3)	−13	−2.90
	tpx	repression	—	—	—

glmZ	glmS	activation	(−40)..(−22)	−19.2	+26.23

ryhB	shiA	activation	(−59)..(−48)	−19.2	*±*1
	ufo/fur	repression	(−31)..(−18)	−13.1	−2.99
	cysE	repression	(−11)..+8	−27.0	−3.00
	frdA	repression	(−17)..+3	−24.5	−2.97
	iscS	repression	(−26)..+2	−23.7	−2.92
	dadA	repression	+9..+39	−29.2	−3.01
	sodB	repression	(−)21..+4	−18.8	−3.00
	sdhC	repression	(−28)..(−8)	−17.1	−2.87

**Table 3:** The modeled changes in translation initiation rate. 65 ncRNA from *E.coli* were tested against rpoS mRNA. Six show a fold change greater than *±*2. The table is sorted in ascending order according to their *Hybridization Energy*.

sRNA	mRNA	Position (mRNA)	Hybridization Energy	Fold Change *α*
dsrA	rpoS	−126..−97	−33.0	+3.0
rprA	rpoS	−133..−94	−30.7	+2.1
arcZ	rpoS	−105..−81	−23.3	+13.5
omrA	rpoS	−27..−9	−21.3	−2.8
ryjA	rpoS	−22..−8	−17.4	−2.8
oxyS	rpoS	+17..+27	−13.1	−2.8

**Table 4:** Gene Ontology term enrichment analysis of 467 genes that appeared to be potentially influenced by RyhB. The analysis was performed with DAVID. The gene list is highly enriched with genes associated with the GO terms *anaerobic respiration* and *iron ion binding*. The *p-value* expresses the likelihood of the observed enrichment happening by chance. *Count* and *%* give the number of genes and the percentage of the whole list of 467 genes associated with the corresponding GO term.

GO name space	GO Term	Count	%	*p*-value
biological process	GO:0006091 generation of precursor metabolites and energy	49	10.5 %	1.0 *×* 10^−12^
biological process	GO:0009061 anaerobic respiration	22	4.7 %	9.5 *×* 10^−12^
molecular function	GO:0043169 cation binding	96	20.6 %	2.5 *×* 10^−10^
molecular function	GO:0046872 metal ion binding	94	20.2 %	2.8 *×* 10^−10^
molecular function	GO:0043167 ion binding	96	20.6 %	3.4 *×* 10^−10^
molecular function	GO:0005506 iron ion binding	45	9.7 %	7.3 *×* 10^−10^
