# Supplementary material for: Modelling Translation Initiation under the Influence of sRNA
Source: Int J Mol Sci. 2012 Nov 30;13(12):16223–40. doi: 10.3390/ijms131216223 (PMC3546686; doi:10.3390/ijms131216223)
Supplement: Supplementary file 1 [file ijms-13-16223-s001.pdf]

Supplementary File

1. Case Study–Effect of RyhB on potH (b0856)

From the 467 genes which show a change in translation initiation rate according to our model, 431 genes are down-regulated and 36 genes show an up-regulation upon RyhB binding. We chose one of those, potH, to illustrate the details of our model results.

RyhB can bind the potH mRNA from position -49 to -13 nt in front of the translation start site, with an hybridization energy of $-35.2\ {}^{kcal}/_{mol}$ and a ΔG of $-12.47\ {}^{kcal}/_{mol}$. After sRNA binding the resulting restructuring of the mRNA leads to a reduced opening energy of the RDS (see Figures S1 and S2).

Figure S1. Change of the opening energy ΔG $[{}^{kcal}/_{mol}]$ along the mRNA due to the binding of RyhB to the potH mRNA. The blue area marks the region where the sRNA binds the mRNA (-49 to -13 nt). The red area marks the ribosome docking site (RDS: -9 to $+21$ nt). The blue and the red line show the opening energy for the unaffected and the sRNA affected mRNA, respectively. Each value expresses the energy needed to open the preceding 30 nt. The black vertical dashed line marks the translation start site. The position is counted from the translation start point.

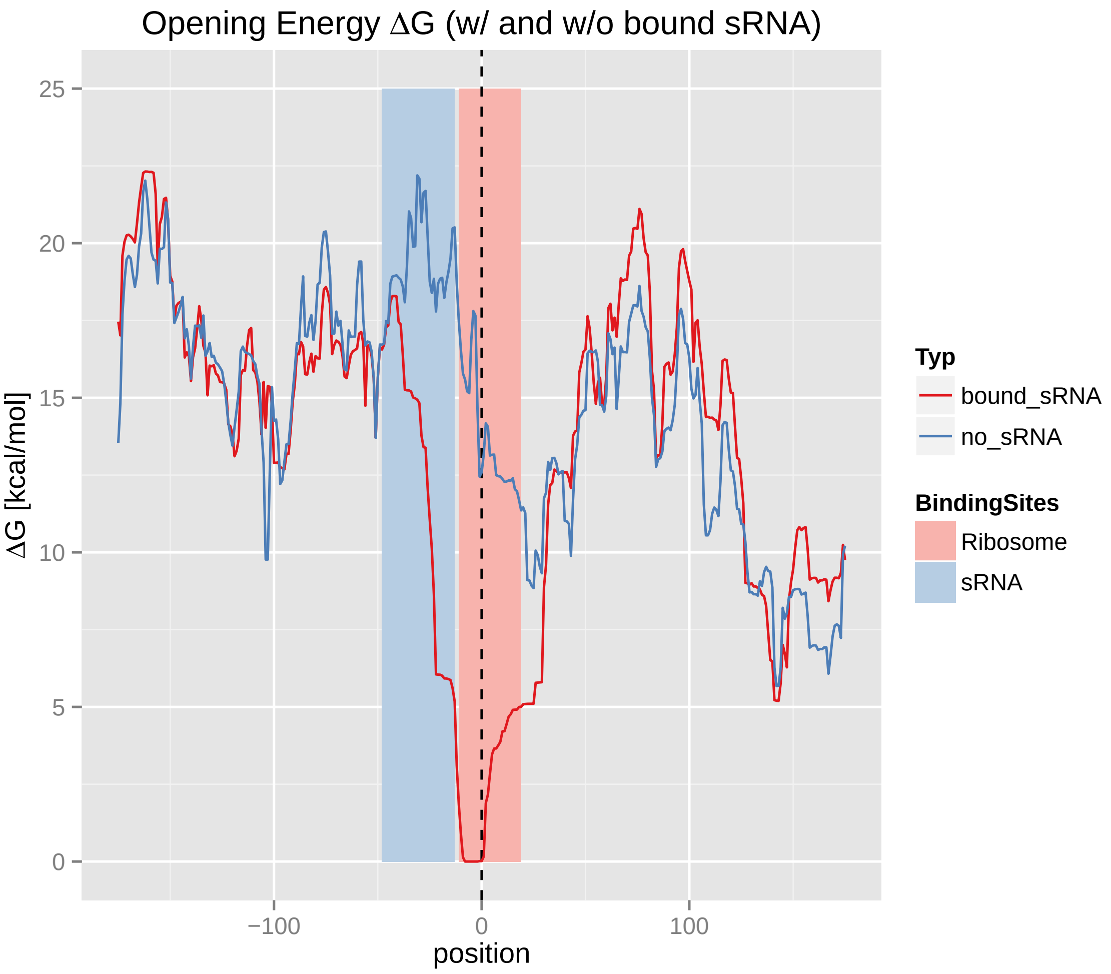

Figure S2. Minimum free energy structure of the translation initiation region without (**A**) and with (**B**) bound RyhB. The sRNA binds in the blue marked region. The black marked sequence stretch represents the ribosome docking site (RDS), with its integrated ribosome recognition site (RRS; marked red). Each base is colored according to the accessibility. From purple to red increases the accessibility of the bases. Plot (**A**) shows that almost the complete RDS is bound inside a very stable hair-pin. After sRNA binding and subsequent refolding, the largest part of the RRS is unfolded in the mfe structure and the rest of the RDS is incorporated in a very loose hairpin (indicated by the red color).

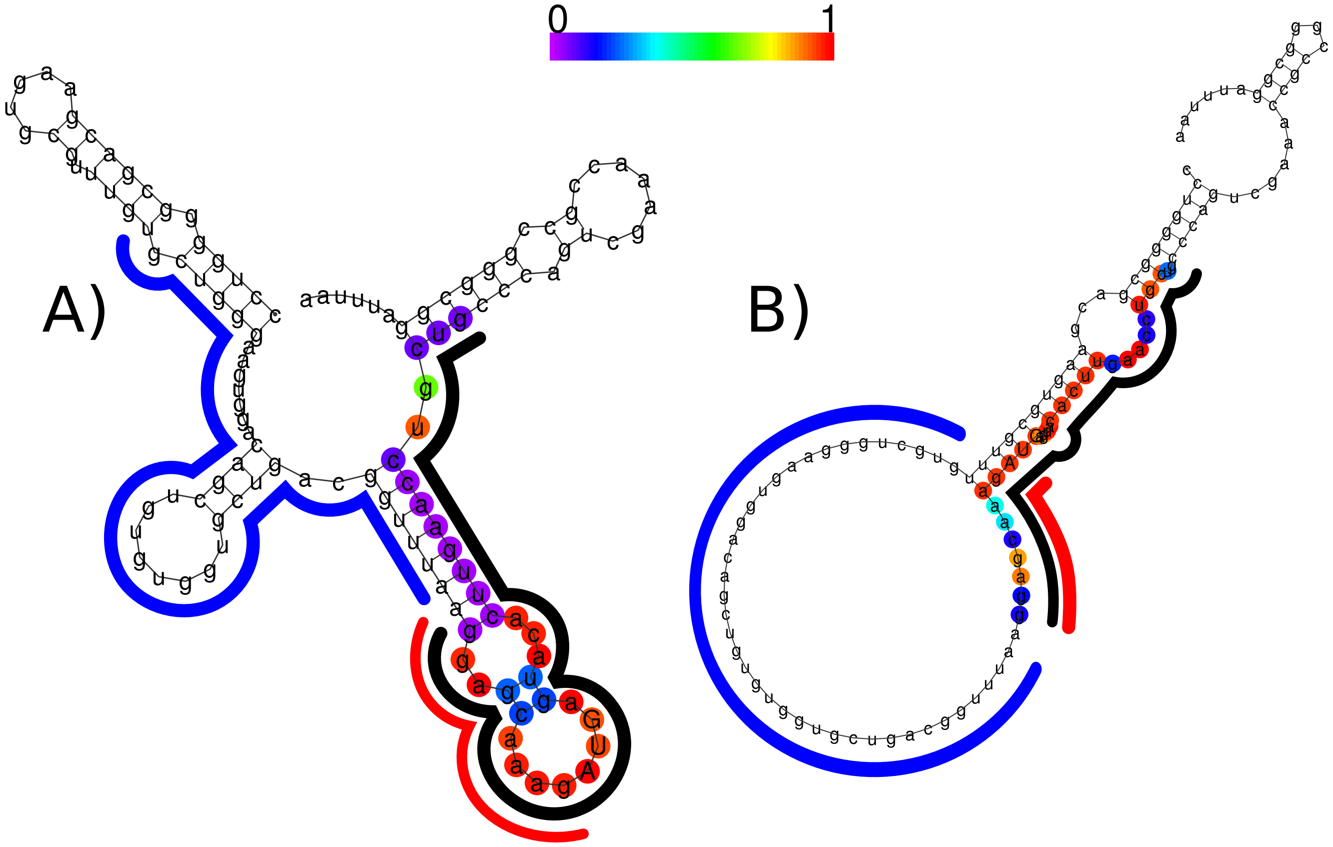

2. Derivation for the Main Equations

The derivation for the two main equations. Namely, the relation between the number of ribosome bound mRNA in dependency of total mRNA m_T, total sRNA s_T, total ribosome r_T and a correction factor n to account for the ribosome occupancy, the reaction constants K_R and K_S, the probabilities to expose the ribosome docking site (RDS) P_{EF} and P_{ES}.

The derivation was done separately for the case where sRNA binding-site overlaps with the ribosome docking site and for the case where those two are spatially separated.

All calculation were performed with *Wolfram Mathematica* (version 8.0). Text in `monospace` revere to Mathematica commands.

2.1. Overlap of RDS and sRNA Binding Site

2.1.1. Basic Equations

sRNA bound mRNA m_S in thermodynamic equilibrium

$$K_S\, m_F\, s_F - m_S = 0 \tag{S1}$$

total sRNA s_T

$$s_T - s_F - m_S = 0 \tag{S2}$$

total mRNA m_T

$$m_T - m_F - m_S - m_R = 0 \tag{S3}$$

ribosome bound mRNA m_R in thermodynamic equilibrium

$$K_R\, m_F\, P_{EF}\, r_F - m_R = 0 \tag{S4}$$

total ribosomes r_T

$$r_T - r_F - n\, m_R = 0 \tag{S5}$$

2.1.2. Elimination of Unknown Variables from Equation System

`Eliminate` s_F, m_F, r_F and m_s from Equations S1–S5 and `FullSimplify`

$$\begin{aligned}(m_R\,(-1 + K_R\,(m_R - m_T)\,n\,P_{EF}) + K_R\,(-m_R + m_T)\,P_{EF}\,r_T)\\ (K_S\,m_R + K_R\,P_{EF}\,(-m_R\,n + r_T)) - K_R\,K_S\,m_R\,P_{EF}\,(-m_R\,n + r_T)\,s_T = 0\end{aligned} \tag{S6}$$

2.1.3. Standard Polynomial Form

Equation S6 is a cubic polynomial equation. Expressed in its standard polynomial form in can be written as

`Collect` m_R in Equation S6 and `FullSimplify` each term on its own

$$\begin{aligned}K_R^2\,m_T\,P_{EF}^2\,r_T^2 - K_S\,(1 + K_R\,P_{EF}\,(m_T\,n + r_T - n\,s_T))\\ +\boldsymbol{m_R}\,(-K_R\,P_{EF}\,r_T\,(1 + K_R\,P_{EF}\,(2\,m_T\,n + r_T) + K_S\,(-m_T + s_T)))\\ +\boldsymbol{m_R^2}\,K_R\,n\,P_{EF}\,(1 + K_R\,P_{EF}\,(m_T\,n + 2\,r_T))\\ +\boldsymbol{m_R^3}\,K_R\,n\,P_{EF}\,(K_S - K_R\,n\,P_{EF})\\ = 0\end{aligned} \tag{S7}$$

2.2. No overlap of RDS and sRNA Binding-site

2.2.1. Basic Equations

sRNA bound mRNA in thermodynamic equilibrium m_S

$$K_S\, m_F\, s_F - m_S = 0 \tag{S8}$$

total sRNA s_T

$$s_T - s_F - m_S - m_{SR} = 0 \tag{S9}$$

total ribosomes r_T

$$r_T - r_F - n\,(m_R + m_{SR}) = 0 \tag{S10}$$

total mRNA m_T

$$m_T - m_F - m_S - m_R - m_{SR} = 0 \tag{S11}$$

ribosome bound mRNA in thermodynamic equilibrium m_R

$$K_R\, P_{EF}\, m_F\, r_F - m_R = 0 \tag{S12}$$

ribosome and sRNA bound mRNA in thermodynamic equilibrium m_{SR}

$$K_R\, P_{ES}\, m_S\, r_F - m_{SR} = 0 \tag{S13}$$

translational active mRNA m_{TA}

$$m_{TA} = m_R + m_{SR} \tag{S14}$$

2.2.2. Elimination of Unknown Variables from Equation System

`Eliminate` m_S, m_F, s_F, r_F, m_{SR} and m_R, from Equations S8 and S14 and `FullSimplify`

$$\begin{aligned} &K_S\,(m_{TA}\,(-1 + K_R\,(m_{TA} - m_T)\,n\,P_{ES}) + K_R\,(-m_{TA} + m_T)\,P_{ES} \\ &r_T)\,((-1 + K_R\,P_{ES}\,(m_{TA}\,n - r_T))\,(m_{TA}\,(-1 + K_R\,(m_{TA} - m_T)\,n \\ &P_{EF}) + K_R\,(-m_{TA} + m_T)\,P_{EF}\,r_T) - K_R\,(P_{EF} - P_{ES})\,(m_{TA}\,n \\ &-r_T)\,s_T) - K_R\,(P_{EF} - P_{ES})\,(m_{TA}\,n - r_T)\,(m_{TA}(-1 + K_R\,(m_{TA} \\ &-m_T)\,n\,P_{EF}) + K_R\,(-m_{TA} + m_T)\,P_{EF}\,r_T) = 0 \end{aligned} \tag{S15}$$

2.2.3. Standard Polynomial Form

Equation S15 is a quintic polynomial equation. Expressed in its standard polynomial form it can be written as

`Collect` m_{TA} in Equation S15 and `FullSimplify` each term on its own

$$
\begin{aligned}
&K_R^2\, m_T\, r_T^2\,(P_{EF}\,(P_{EF}-P_{ES}\,(1+Ks\,(m_T+K_R\,m_T\\
&\quad P_{ES}\,r_T)))+Ks\,(P_{EF}-P_{ES})\,P_{ES}\,s_T)\\
\\
&+\boldsymbol{m_{TA}}\,K_R\,r_T\,(P_{ES}-K_R\,P_{EF}^2\,(2\,m_T\,n+r_T)\\
&+P_{EF}\,(-1+K_R\,P_{ES}\,(2\,m_T\,n+r_T)+Ks\,m_T\,(1+K_R\,P_{ES}\\
&\quad(r_T\,(3+2\,K_R\,P_{ES}\,r_T)+m_T\,n\,(2+3\,K_R\,P_{ES}\,r_T)))\\
&-Ks\,(1+K_R\,P_{ES}\,(2\,m_T\,n+r_T))\,s_T)+Ks\,P_{ES}\\
&\quad(s_T+K_R\,P_{ES}\,r_T\,s_T+m_T\,(1+K_R\,P_{ES}\,(r_T+2\,n\,s_T))))\\
\\
&+\boldsymbol{m_{TA}}^2\,(K_R\,n\,(P_{EF}-P_{ES})\,(1+K_R\,P_{EF}\,(m_T\,n+2\,r_T))\\
&+Ks\,(-1+K_R\,(-K_R\,m_T^2\,n^2\,P_{EF}\,P_{ES}\,(1+3\,K_R\,P_{ES}\,r_T)-r_T\,(P_{EF}\\
&\quad(1+K_R\,P_{ES}\,r_T)^2+P_{ES}\,(2+K_R\,P_{ES}\,r_T))-m_T\,n\,(P_{ES}\\
&+2\,K_R\,P_{ES}^2\,r_T+P_{EF}\,(1+6\,K_R\,P_{ES}\,r_T\,(1+K_R\,P_{ES}r_T)))\\
&+n\,(P_{EF}-P_{ES})\,(1+K_R\,P_{ES}\,(m_T\,n+2\,r_T))\,s_T)))\\
\\
&+\boldsymbol{m_{TA}}^3\,K_R\,n\,(K_R\,n\,P_{EF}\,(-P_{EF}+P_{ES})\\
&+Ks\,(P_{EF}\,(1+K_R\,P_{ES}\,(K_R\,m_T^2\,n^2\,P_{ES}+r_T\,(4+3\,K_RP_{ES}\,r_T)\\
&+3\,m_T\,(n+2\,K_R\,n\,P_{ES}\,r_T)-n\,s_T))+P_{ES}\,(2+K_R\,P_{ES}\\
&\quad(2\,r_T+n\,(m_T+s_T)))))\\
\\
&+\boldsymbol{m_{TA}}^4\,(-K_R^2\,Ks\,n^2\,P_{ES}\,(P_{ES}+P_{EF}\,(2+K_R\,P_{ES}\,(2\,m_T\,n+3\,r_T))))\\
\\
&+\boldsymbol{m_{TA}}^5\,K_R^3\,Ks\,n^3\,P_{EF}\,P_{ES}^2\\
\\
&=0
\end{aligned}
\tag{S16}
$$
